# Induction of calcification by serum depletion in cell culture: a model for focal calcification in aortas related to atherosclerosis

**DOI:** 10.1186/1476-511X-7-2

**Published:** 2008-01-29

**Authors:** Howard HT Hsu, Antonio Artigues, Maria T Villar

**Affiliations:** 1 Department of Pathology and Laboratory Medicine, University of Kansas Medical Center, Kansas City, Kansas, 66160, USA; 2 Department of Molecular Biology and Biochemistry, University of Kansas Medical Center, Kansas City, Kansas, 66160, USA

## Abstract

**Background:**

Since aortic calcification has been shown to initiate in the lower zone of well-thickened plaques (LZP) adjacent to the aortic media of rabbits fed supplemental cholesterol diets, a restricted supply of serum to vascular cells could play a role in vascular calcification. This study was designed to use a cell culture model to support this hypothesis.

**Results:**

Rabbit aortic smooth muscle cells were grown to confluence in a culture media containing 10 % fetal bovine serum (FBS). The confluent cells were then exposed to the media for 2 hrs with or without serum at a Ca × P ion product range of 4.5–9.4 mM^2^. In contrast to the cells cultured in the presence of FBS, confluent cells in its absence displayed marked mineral-positive alizarin red staining and infrared absorption of mineral phosphate. A kinetic parameter C_1/2 _was used to designate the concentration of serum or its protein constituents needed to reduce the deposition of Ca and P by half. The C_1/2 _for FBS and rabbit serum was 0.04–0.07 % The C_1/2 _value for rabbit serum proteins was 13.5 μg/ml corresponding to the protein concentration in 0.06 % of serum. This C_1/2 _was markedly smaller than 86.2 μg/ml for bovine serum albumin present in 0.37 % serum (p < 0.05). Serum depletion also caused marked membrane translocation as evidenced through a specific apoptosis dye uptake by cells. The proteomic analysis of calcifying vesicles, which can be released by serum depletion, revealed several calcification-related proteins.

**Conclusion:**

The aortic smooth muscle cell culture model suggests that serum depletion may play a role in the initiation of aortic calcification. The serum exhibits remarkable ability to inhibit cell-mediated calcification.

## Background

Clinical and epidemiological studies have implicated vascular calcification in myocardial infarction [1], instability and rigidity of the arterial wall [2], bioprosthetic valve failures [3], etc. In spite of the evidence that both physiological and pathological calcification is regulated through gene expressions of osteopontin (OPN) [4], matrix γ-carboxyglutamate protein (MGP) [5], and osteoprotegerin (OPG) [6], the mechanisms whereby calcium phosphate minerals are initially deposited in the arterial wall remain uncertain [7-9]. The issue of the involvement of the active process vs. passive process in atherosclerosis-related calcification was reviewed by Schinke et al. [8]. This issue can be further complicated by the difficulty in separating the effects of the agents on nascent mineralization from those on mineral proliferation. The paradoxical nature of different hypotheses could stem from the lack of detailed information of the specific molecular events aiming at the earliest stage of calcification. The development of strategies for arresting the initial stage of calcification could be a crucial step in the prevention of further spread of unwanted calcification. One of the underlying mechanisms of vascular calcification was proposed to be tightly linked to osteogenesis [10,11], since bone formation and calcification in human aortas were first described by Virchow in 1863 [12] and later shown to be in human cardiac valves by Mohler and colleagues in 2001 [13] both at the advanced stages of atherosclerosis. However, on the basis of genetic analyses and the rare occurrence of bone formation in human lesions, Schinke et al [8,9] conceptualized the onset of vascular calcification as being related to a dystrophic process independent of osteogenesis. An alternative hypothesis for the participation of a remote bone resorption process in vascular calcification was advocated by Price and colleagues using hypervitamin D-treated rat model [14]. This model was deduced from the observation that subcutaneous injections of specific bone resorption inhibitors such as bisphosphonates to the rat inhibited calcification in arterial media. Another interesting observation that may also underlie the cause of vascular calcification in human subjects was the finding of mineral-associated nanobacteria-like structures in advanced atherosclerotic aortic walls [15].

**Figure 1:**
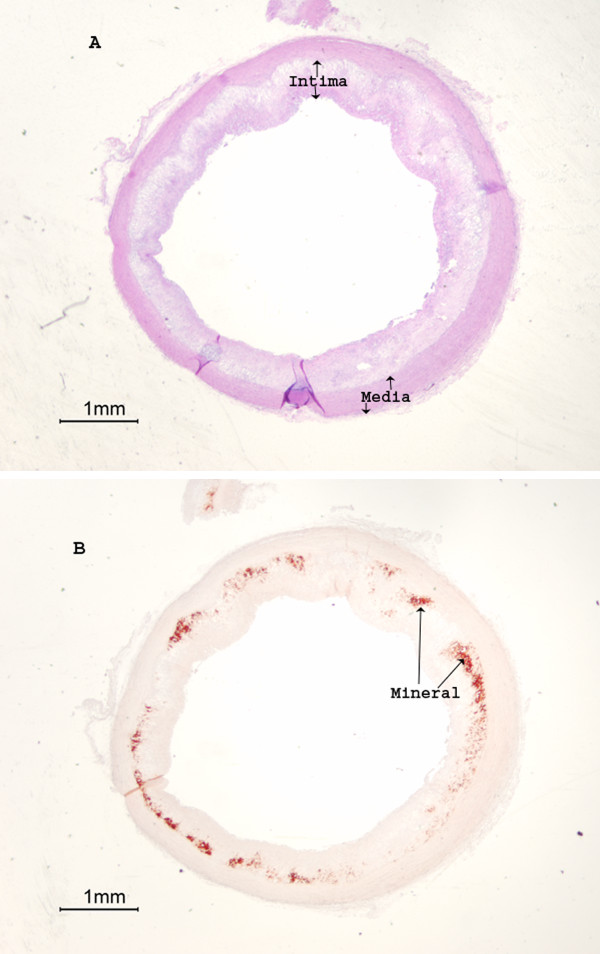
Rabbit aortic calcification occurred in the lower zone of thickened plaques adjacent to the media. The normal control contained neither thickened intima nor calcification in the media (not shown) [see Refs 7, 19]. Rabbits were fed an atherogenic diet containing 0.5% cholesterol and 2% peanut oil for 4 months. Segments of thoracic aortas were fixed with formalin, paraffin blocked, thin sectioned. A) H & E staining was used to show intimal thickening. B) Mineral deposits were visualized as alizarin red stains (AR).

Numerous studies indicated that calcification in rabbit aortas can be induced by cholesterol supplemental diets [16,17]. Previous observations in this laboratory demonstrated that thoracic aortic calcification in rabbit fed cholesterol supplemental diets was not initiated randomly; rather the calcification started in the lower zone of extensively thickened plaques (LZP) adjacent to the surrounding media, and progressed along this interface [7,18,19] (for illustration, see Fig. 1 in this section). The apparent lack of complex bone structures including osteoid, osteoblasts, and notably alkaline phosphatase activity (an obligatory bone and cartilage biomarker [20]) in the LZP suggests that the initiation of calcification in rabbit thoracic aortas induced by cholesterol supplemental diets is independent of the osteogenic process [7,18,19,21,22]. Since juvenile rabbits were selected for those studies, the role of remote bone resorption in LZP calcification would be minimal. The aspect of LZP calcification resulting from the infection by nanobacteria in rabbit aortas is difficult to determine because of the size similarity between calcifying vesicles and mineral-associated nanobacteria-like particles [15]. Alternatively, the initiation of calcification in the LZP suggests that the restricted supply of blood to the vascular cells implemented by thickened plaques may play a significant role in calcification. To further support the hypothesis that serum depletion in LZP may cause calcification, a cell culture model is herein reported to determine whether deprivation of serum from culture media may induce calcification in rabbit aortic smooth muscle cell culture. The *in vitro *experiments in the present study suggest that aortic calcification is related to dystrophic processes independent of osteogenesis.

## Results

### Induction of calcification *in vitro *by serum depletion

**Figure 2:**
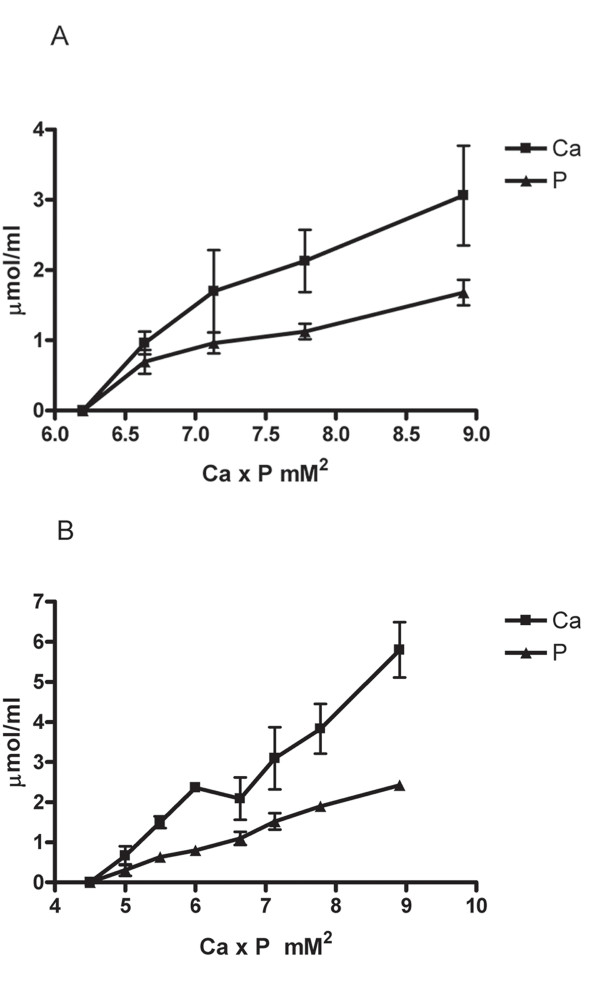
Effect of Ca × P ion products on serum depletion-induced calcification. Confluent cells were incubated in DMEM without serum at various levels of Ca × P ion products for 2 hrs. The culture dishes were washed twice with TBS and then suspended in 0.5 ml 0.1 N HCl. The extracts were centrifuged in a microfuge for 10 min at the top speed to collect the supernatants. Each set of three replicate experiments covered the indicated range of ion products. Calcification in cell culture was expressed as μmol of Ca and P deposited per ml of 0.1 N HCl extracts. Each data point for Ca and P represents Mean ± S.D. calculated from the average of duplicates in each experiment. The contributions of endogenous Ca and P in serum and the culture media to the ion products were all taken into considerations. (A) Ca concentration was kept at 1.80 mM whereas various concentrations of P were used. (B) P concentration was kept at 1.87 mM whereas various concentrations of Ca were used.

**Figure 3:**
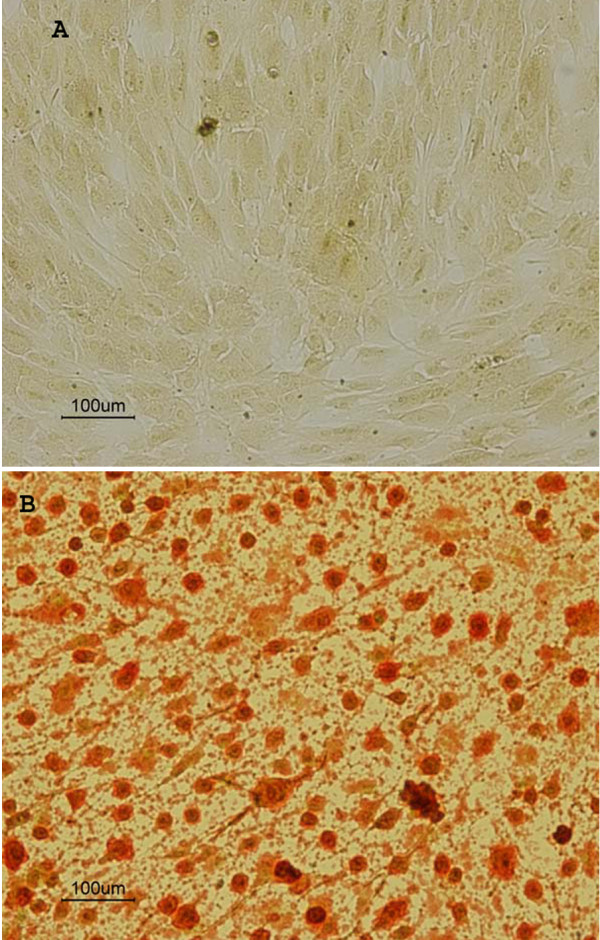
Alizarin red mineral staining of smooth muscle cell cultures exposed to DMEM with or without 10% FBS for 2 hrs. A) Cells exposed to DMEM containing 10% FBS. B) Cells exposed to DMEM without serum. The initial Ca × P ion product in culture media was 9.37 mM^2^.

**Figure 4:**
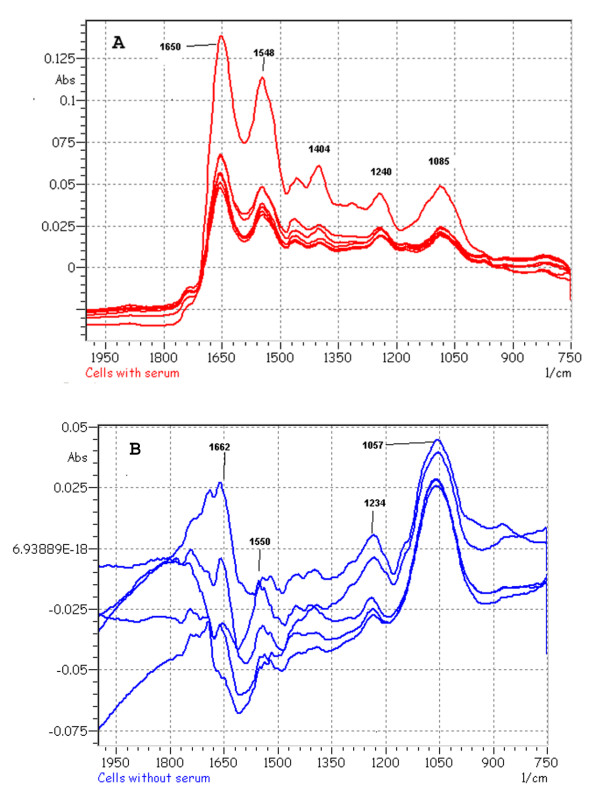
Fourier-transform infrared microspectroscopic patterns of mineral deposits. A) Cells exposed to modified DMEM with 10 % rabbit serum for 2 hrs. B) Cells exposed to the media without rabbit serum. After a 2-hr exposure to the media with or without the serum, cells were washed twice with TBS, twice with water, scraped off from the dishes, and finely suspended with Pasteur pipets. The initial Ca × P ion product in culture media was 9.37 mM^2^. Five-μl aliquots containing 1 μg proteins were then spotted onto BaF_2 _windows for infrared analysis. The infrared spectra were obtained by scanning the deposit over a 0.6–0.8 cm path. The graphs show the intensity of protein amide at wavenumber 1650 and a minute peak of mineral P appearance at wavenumbers 1050–1080 from the control with serum exposure whereas the P peak was much more prominent in the serum-depletion experiments. A Shimadzu Fourier-transform infrared microspectroscopy model AIM8400/8800 was used for the analysis.

To test the hypothesis that serum restriction in the lower zone of plaques initiates focal calcification [7,18,19], we used a cell culture model to determine whether calcification in rabbit aortic smooth muscle cell culture could be induced by the deletion of serum from the culture media. We first determined the minimal range of Ca × P ion products in the culture media needed to initiate calcification in cell culture without serum. When Ca concentration was maintained initially at 1.80 mM Ca (calcium ions) with varying concentrations of P (phosphate ions) in the media, the colorimetric measurements indicated that calcification increased with ion products and that a minimum of 6.7 mM^2 ^was required to initiate calcification (Fig. 2A). When phosphate concentration was maintained at 1.80 mM with varying concentrations of Ca the minimal ion product was found to be 5.0 mM^2 ^significantly lower than 6.7 mM^2 ^(p < 0.05, Fig. 2B). We also used a specific alizarin red staining for Ca minerals to study the effect of serum depletion on calcification. In the presence of 10% FBS or normal rabbit serum in the culture media, cells did not exhibit alizarin stains (Fig. 3A). In contrast, serum depletion caused the deposits of minerals with marked staining (Fig. 3B). The mineralization in the absence of serum was further confirmed by the Fourier-transform infrared microspectroscopic analysis (FT-IR). The peaks of mineral P at the wavenumber ranges of 1050–1080 relative to the protein amide peak of 1650 from the extracts of the cells exposed to culture media with serum (Fig. 4A) were much smaller than those without serum (Fig. 4B). The FT-IR also indicates that the amide peak pattern after mineralization was much less defined than the pattern before calcification.

**Figure 5:**
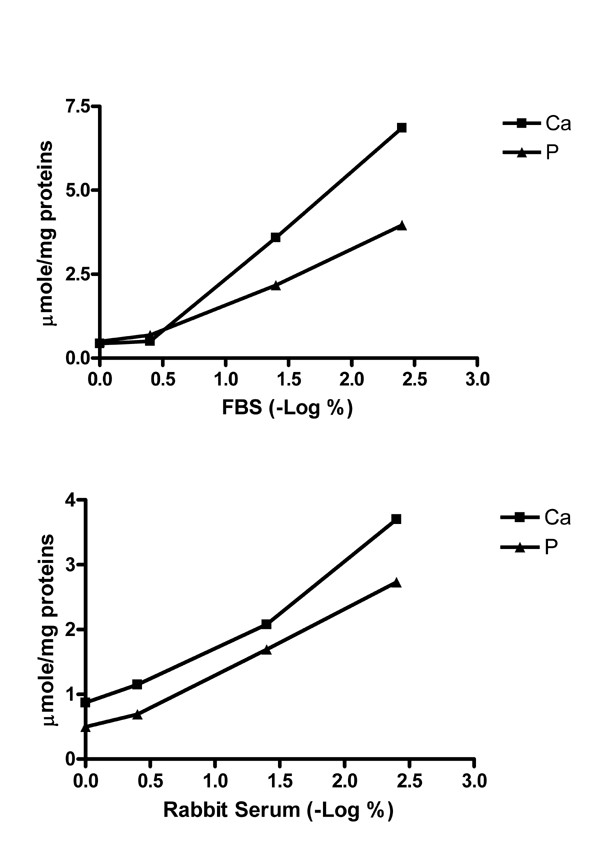
Effects of the concentration of FBS or rabbit serum on the prevention of calcification in cell culture. Confluent cells were exposed to various concentrations of FBS or rabbit sera for 2 hrs. Concentrations of the sera ranging from 0.004 to 1% were used. The initial Ca × P ion product in culture media was 9.37 mM^2^. Each data point for Ca and P determinations represents the average of duplicate data. The C_1/2 _values in these experiments were about 0.07 % for Ca and P deposition. The serum levels are expressed as a negative log scale of the percentage of the serum, i.e., the higher the scale to the right of the X axis, the more the serum was diluted as indicated. The deposition of Ca and P was determined by colorimetric procedures and expressed as μmol Ca or P per mg of cell proteins. At 0.004% serum, the amounts of mineral Ca and P were nearly equal to those without serum. (A) FBS. (B) Normal rabbit serum.

**Figure 6:**
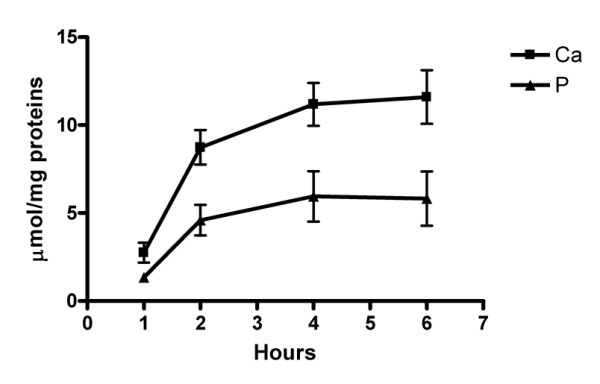
Time course of serum depletion-induced calcification. Calcification was expressed as μmol of Ca and P deposited per mg of cell proteins. Confluent cells were incubated in DMEM containing 0.07 % rabbit serum for various time intervals. Each set of three replicate experiments contained 4 dishes to include all time intervals. Data at each time point represent Mean ± SD. For Ca and P assay, duplicate determinations were run to obtain the average for statistical analysis. The initial Ca × P ion product in culture media was 9.37 mM^2^.

Fig. 5 shows that the deposition of Ca and P minerals as assessed by colorimetric assay decreased with an increase in the concentration of FBS or rabbit sera in media. A time course study demonstrated that serum depletion caused significant calcification in a time-dependent manner, which indicated that it took 1.5 hr (t _1/2_) to reach half maximal activity at a Ca × P ion product of 9.37 mM^2 ^(Fig. 6). At t _1/2 _about 11.0 ± 3.1 μmol Ca and 7.1 ± 1.1 μmol P per mg of cell proteins were deposited.

**Figure 7:**
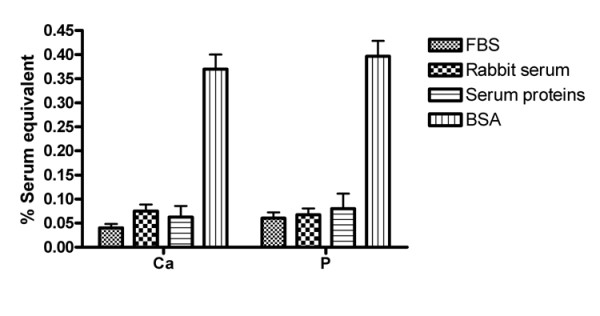
Comparison of the effectiveness of FBS, rabbit sera, BSA, and serum proteins in the prevention of calcification in cell culture. The efficacy of proteins and sera to inhibit calcification is expressed as C_1/2 _(Y-axis), representing a parameter for the concentration of serum needed to inhibit one half of serum depletion-induced calcification. For the serum protein assessment, the C_1/2 _values were normalized to the percentage of the serum on the basis of their concentrations in the serum. The C_1/2 _values were obtained from 3 independent experiments with sera or proteins precipitated by 60% ethanol. One-Way ANOVA analysis was used to determine statistical significance among these parameters. No significance was noted except that the C_1/2 _from BSA was markedly higher than those from sera or total serum proteins, meaning that BSA was much less effective than others (p < 0.05). The initial Ca × P ion product in culture media was 9.37 mM^2^.

A kinetic parameter C_1/2_, which represents the concentration of serum or serum protein factors needed to reduce serum depletion-induced calcification by half after 2 hrs of incubation, was used to compare the effectiveness and specificity of different sera and serum factors in calcification prevention. All of the following experiments were conducted at the Ca × P ion product of 9.37 mM^2 ^to maximize the serum depletion effect for comparison. Fig. 7 shows that the C_1/2 _for FBS in Ca and P deposition experiments was 0.04 ± 0.01% insignificantly different from 0.07 ± 0.03% for rabbit serum (p > 0.05). The C_1/2 _values for serum proteins isolated by ethanol precipitation and those for bovine serum albumin (BSA) were 13.5 ± 8.6 and 86.2 ± 12.3 μg/ml, respectively, corresponding to the concentrations of serum proteins and albumin in 0.06 ± 0.04 and 0.37 ± 0.05% of rabbit serum, respectively. Similar patterns for C_1/2 _were seen with P deposition experiments.

**Figure 8:**
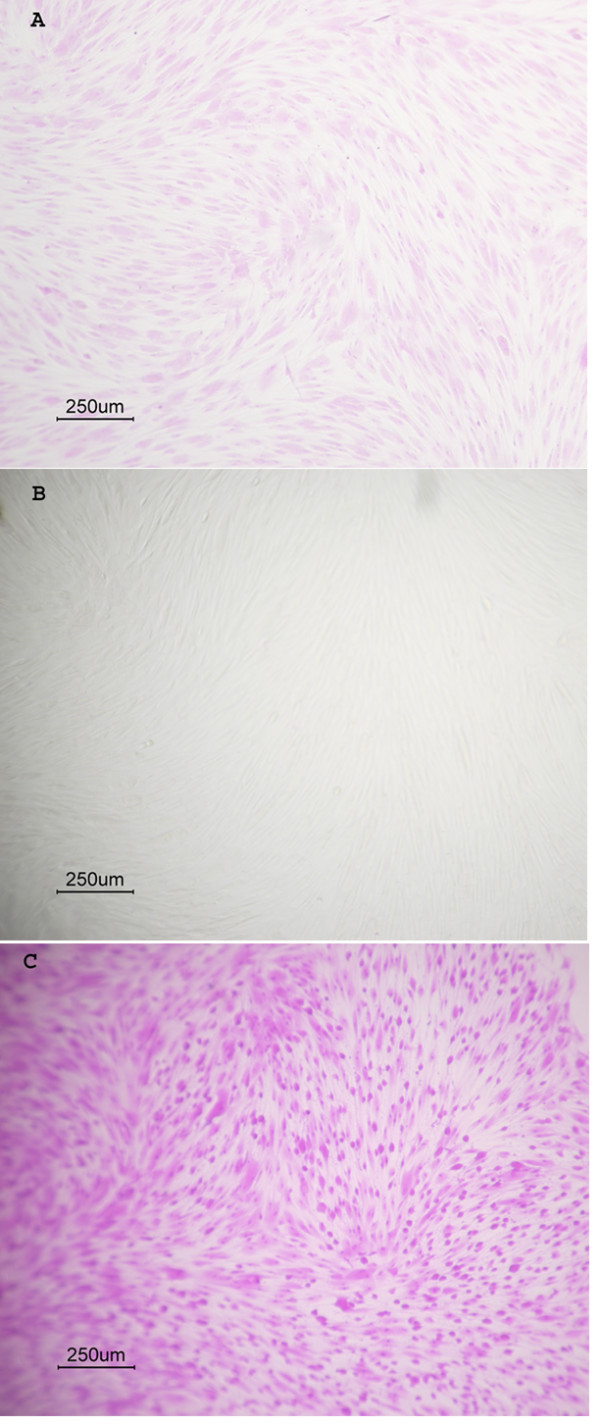
Induction of membrane translocation by serum depletion. The apoptosis-related membrane translocation assay was performed using a commercial kit (Accurate Chemical Scientific Corp.), which measured the uptake of a specific dye by cells as a result of apoptosis-induced membrane phosphatidylserine and phosphatidylcholine translocation. The assay was performed in 96-well microtiter plates. Cells were grown to confluence and followed by thoroughly washing with twice with PBS to remove serum, and then exposed to media with or without 10% serum for 1 1/2 hrs. At the end point, each well was washed twice with PBS and then exposed to the apoptosis dye reagent in DMEM with or without 10% FBS for 30 min. The cells were then photographed after the removal of dyes and then followed by PBS washing. (A) Control cells exposed to DMEM-10% FBS and 5 mM H_2_O_2 _for 1 1/2 hrs and followed by a 30 min-exposure to the same media containing the apoptosis marker dye. (B) Cells exposed to serum-free media for 1 1/2 hrs and then culture media containing both 10% FBS and the apoptosis dye marker for 30 min. (C) Cells exposed to serum-free media for 1 1/2 hrs and then serum-free media containing the apoptosis dye marker for 30 min.

Since mineral-associated apoptotic bodies with size similar to vesicles were found in calcified aortas [23], we determined whether serum depletion may induce calcification through apoptosis. We used the uptake of a specific apoptosis dye agent by cells to show membrane translocation, an early event of apoptosis [24]. The treatment of cells with the media containing both 10% serum and 5 mM hydrogen peroxide for 1 hr caused more than 90% of cells to undergo membrane translocation (Fig. 8A). A further 2-hr exposure of the affected cells to media with 10 % serum after the removal of 5 mM hydrogen peroxide exhibited neither detectable AR mineral stains nor colorimetric quantities of Ca or P deposition (not shown). The exposure of cells to the media with or without serum for 1 1/2 hrs and followed by incubations with the membrane translocation marker dye in the media containing 10% FBS for 30 min did not take up the dye (Fig. 8B). In contrast, the cells displayed marked membrane translocation only if FBS was omitted from the dye-containing media after 1 1/2 hr exposure to serum-free media (Fig. 8C).

To further support the role of calcifying vesicles in LZP calcification, the protein genomic analysis was performed on these vesicles, which were shown to be released by serum depletion [7], to identify calcification-related proteins that may increase local concentrations of Ca and P in favor of calcification. The data in Table 1 demonstrate an array of calcification-related proteins in the vesicles. The identified proteins included calpactins (also known as annexins) [25], calreticulin [26], integrin [27], fibrillin [28], Ca-Mg-ATPase [21,22], and ATP synthase (may provide ATP for phosphate production).

**Table 1:** List of identified proteins related to calcification in calcifying vesicles from tadem spectrometry. Data were analyzed using a Biowors 3.2 (ThermoFinnigan) program.

Proteins	Peptide Sequence
Calpactin I Heavy chain	SNAQRQDIAFAYQR (2, 4.50, 0.32) LSLEGDHSTPPSAYGSVK (2, 4.00, 0.40) RAEDGSVIDYELIDQDAR (2, 3.69, 0.29) GVDEVTIVNILTNR (1, 2.83, 0.29)
Calreticulin or CRP55	FYALSAR (1, 2.00, 0.44) FKPFSNKGQPLVVQFTVK (2, 3.75, 0.64) EPVVYFKEQFLDGDGWTER.G (2, 10.2, 0.54)
Integrin beta 1	SKGTAEKLQPEDITQIQPQQLVLQLR (1, 2.02, 0.36) GTAEKLQPEDITQIQPQQLVLQLR (2, 5.42, 0.54) LQPEDITQIQPQQLVLQLR (2, 5.42, 0.54) SGEPQTFTLK (2, 2.02, 0.36)
Fibrillin	IKGTQC#EDINEC#EVFPGVC#K (2, 3.00, 0.36) IKGTQC#EDIDEC#EVFPGVCKNGLCVNTR (3, 4.58, 0.35)
ATPase β subunit	VALTGLTVAEYFRDEEGQDVLLFIDNIFR (3, 2.70, 0.27) AIAELGIYPAVDPLDSTSR (2, 4.76, 0.12)
ATP synthase	ITSTKEGSITSIQAIYVPADDLTDPAPATTFAHLDATTVLSR (3, 5.69, 0.17) VLDSGAPIKIPVGPETLGR (2, 3.81, 0.65) ILQDYKSLQDIIAILGMDELSEEDKLTVSR (3, 3.78, 0.55) ILGADTSVDLEETGRVLSIGDGIAR (2, 3.26, 0.19) TGAIVDVPVGEELLGRVVDALGNAIDGKGPIGSK (2, 5.13, 0.47) AIAELGIYPAVDPLDSTSR (1, 3.0, 0.46)

The following set of criteria for high confidence were used: minimum cross-correlation score of (Xcorr) of 1.9, 2.5 and 3.5 for 1, 2 and 3 charges ions respectively, a delta correlation score (Δcorr) greater than 0.1, and a minimum of 2. These parameters are presented in the parentheses in sequence of charges, Xcorr, and Δcorr. Database used was obtained from the NCBI repository. C# indicates a carboxymethylcisteine.

## Discussion

A previous report in this laboratory demonstrated that unlike the control experiments in which abundant blood vessels with bone marrow were present in Saos-2 osteosarcoma extract-induced ectopic bones in nude mouse skeletal muscles, the lower zone of plaques (LZP) in atherosclerotic rabbit aortas lacked the evidence of vessel intrusions and bone formation [7]. These observations suggest that the induction of LZP calcification could be caused by the restriction in blood supply to the LZP due to intimal thickening without a prerequisite osteogenesis [10,11] or remote bone resorption [14]. This contention was further supported by the present data obtained from the experimentation with cultured rabbit aortic smooth muscle cells exposed to serum depletion. In spite of the present *in vitro *model, factors that underlying the mechanism of the serum depletion-induced LZP calcification remains to be established. A reduced amount or a lack of some serum proteins in the LZP may play a role in LZP calcification. For example, anhydrase and hemoglobin are essential proteins in the regulation of blood pH and their absence could potentially influence pH dependent-mineralization process. The lack of hemoglobin may affect oxygen tension and could in theory alter acid-base chemistry through the isohydric shift, although the role of oxygen in LZP calcification has yet to be established. Moreover, potent mineralization inhibitors such as OPN [29] and MGP [30] in blood could be prevented from reaching LZP by intimal thickening. Other factors such as alterations in osmolarity, which can be affected by serum protein deletion, remain unknown. However, the present observation shows that much less amounts of whole serum proteins than serum albumin were needed to inhibit calcification, suggesting that change in osmolarity by serum depletion may not play a significant role in the calcification. An attempt to reproduce intimal thickening *in vivo *by the addition of cholesterol to SMC culture increased neither cell proliferation nor calcification (not shown). This is expected since aortic calcification in rabbits fed cholesterol diets did not occur until intima was extensively thickened [7,18,19].

The minimal requirement of Ca × P ion products of 5.0 mM^2 ^in excess of the serum level of 3.5 mM^2 ^for serum depletion-induced calcification *in vitro *suggests that additional mechanisms are needed to increase Ca and/or P in the LZP. The current experiments using a sensitive measurement of Ca with Arsenazo III dye [31] indicated that 5.02 ± 0.25 mM Ca (6 serum samples) was present in rabbit serum vs. 3.23 mM using an electrode assay procedure [32]. A protein-free fraction of serum obtained from 10% trichloroacetic acid (TCA) precipitation followed by the removal of TCA through lyophilization yielded the same result (not shown). The precision and accuracy of the procedure was verified by the use of known quantities of calcium carbonate and calcium mono- or dibasic phosphate dissolved in 0.1 N HCl. Although the difference in quantities between the two procedures could be due to the technical issue, the data suggest a large pool of serum Ca available for calcification through the diffusion process. High ion products can be further achieved via the hydrolysis of phosphoesters in cells. High concentrations of β-glycerophosphate ranging from 5–10 mM were routinely used in numerous studies of calcification in cell cultures including bone cells [33] and bovine SMCs [34]. However, the current data indicated that rabbit SMCs cultured for up to 14 day in the presence of 10% FBS and/or 10 mM β-glycerophosphate with a biweekly medium changes did not display calcification (data not shown). This is expected since rabbit SMCs and aortas neither have alkaline phosphatase activity nor the ability to hydrolyze β-glycerophosphate [18]. The paradoxical difference in the effect of serum on calcification between rabbit and bovine SMCs remains to be established. Moreover, the Ca × P ion products needed to initiate mineralization with varying Ca and constant P levels appeared to be lower than those with constant Ca and varying phosphate levels (p < 0.05). The changes in FT-IR of amide peak after mineralization also suggest that the perturbation of protein absorption in the infrared spectrum could be attributable to Ca binding or by overlapping mineral deposits.

We previously reported that serum depletion in culture media increased the production of calcifying vesicles, which contain enzymes capable of hydrolyzing nucleotides including Ca-Mg ATPase, AMPase, ADPase, and nucleoside triphosphate pyrophosphohydrolase [21,22]. The elevated enzyme activities could increase either Ca or P accumulation in favor of mineralization, [7]. The present mass spectral data as shown in Table 1 demonstrate the presence of various calcification-related proteins in calcifying vesicles. These observations, therefore, further support the role of the release of these vesicles in vascular calcification [7,19,21,22]. Furthermore, calcifying vesicles could be trapped and concentrated within LZP by intimal thickening, thereby promoting focal calcification. The ability of serum depletion to induce calcifying vesicles in SMCs is consistent with the observation that calcifying vesicles can be released from human osteosarcoma cells (Saos-2) within 30 min after exposure to serum-free media [35]. Thus, the increased vesicle activity resulting from serum depletion in LZP and the availability of a large pool of serum Ca and/or P diffusible through LZP could provide a mechanism to surpass the threshold ion product needed for calcification.

Apoptotic bodies have been suggested to play a role in physiological and pathological calcification. The present observations of the association between serum-depletion-induced membrane translocation (MT), which is an early event of apoptosis and calcification suggest that the change in membrane topology may play a pivotal role in vascular calcification. Such contention is consistent with our observation that calcifying vesicles were accumulated on the surface of SMCs upon incubation with serum-free culture. The lack of association between peroxide-induced MT and cell-mediated calcification is likely due to the presence of serum in the MT assay, since the MT induced by serum depletion or peroxide can be fully reversed within 30 min by the serum replenishment (data not shown). The less degree of the dye uptake shown in Fig. 8A than in 8B was probably due to a partial inhibition of MT by the inclusion of serum in the assay for peroxide-induced MT. Thus, these data indicate that serum can inhibit both membrane translocation and calcification.

The marked inhibitory effect of extensively diluted serum indicates that minute quantities of some serum protein components play a pivotal role in LZP calcification. It appears from C_1/2 _data that serum albumin may need to synchronize with other serum proteins to produce maximal effect. Despite these experiments, the mechanisms underlying the inhibitory effect of the minute amounts of serum on calcification are unclear. In contrast to the preventive effect of serum on calcification in rabbit SMC culture reported herein, a rat model in which animals were injected with high dosage of vitamin D, the increase in fetuin-mineral complex level in rat serum was shown to correlate with arterial calcification in the media [36]. The paradoxical differences in the effects of the serum on vascular calcification at the two distinct parts of the arteries between these two species are unclear. It is also plausible that some minor proteins in the serum with the ability to inhibit mineralization such as osteopontin (OPN) [29,37], matrix γ-carboxyglutamate (Gla) protein (MGP) [30], and osteoprotegerin (OPG) [38] may play a major role in the serum effect. Giachelli and colleagues [32] demonstrated that OPN at a level of 50 ng/ml inhibited bovine SMCs *in vitro *calcification, which occurred after a 10-day exposure to the media containing 10% serum and 10 mM β-glycerophosphate. Since the effective concentration of OPN in these experiments was 5-fold higher than the serum OPN level [29], whether the serum level of OPN can inhibit serum depletion-induced calcification remains to be established. A direct test to see if OPN or MGP at the serum levels can inhibit calcification in the rabbit aortic SMC short-term cell cultures awaits the availability of purified proteins for testing. The purified recombinant OPN from EMP Genetech did not inhibit SMC calcification (not shown), probably due to insufficient phosphorylation in the recombinant protein. Since a range of 5–11 ng/ml of OPN was present in human sera [29], a comparison of C_1/2 _values among the total serum proteins and regulatory proteins could rule in or out whether one of these proteins is essential for the prevention of unwanted calcification. In addition, whether there is a need of synchronization with other essential proteins can be addressed by the comparison of C_1/2 _values among these proteins.

Altogether, the data from the *in vitro *cell culture experiments herein support the hypothesis that serum depletion in LZP imposed by lesion thickening could trigger calcification through the accumulation of calcifying vesicles on the cell surface [7] and/or the elimination of the potent serum mineralization inhibitors in the LZP. The short-term nature of the effect of serum depletion on calcification in cultures also suggests that the initial events of calcification in rabbit thoracic aortas during dietary cholesterol intervention are independent of osteogenesis and could be related to membrane translocation, an early event in apoptosis [7-9,23].

## Conclusion

The aortic smooth muscle cell culture model suggests that serum depletion may play an important role in the initiation of aortic calcification. The serum exhibits a remarkable ability to inhibit cell-mediated calcification.

## Methods

### Animals, culture reagents, and chemicals

DMEM, FBS, and all reagents for culture were obtained from GIBCO. Normal rabbit serum was a product of Sigma Chemicals Inc. For Ca and P determinations, serum samples were taken from the ear veins of normal rabbits kept in our animal facility center. Arsenazo III dye kit for Ca determination was purchased from Fischer Scientific. A standard phosphate assay kit was a product of Sigma Chemicals. Apoptosis assay kit was purchased from Accurate Chemical Scientific Corp. BaF_2 _windows for Fourier transform infrared microspectroscopic analysis were obtained from Spectra Tech. Rabbits were purchased from Myrtle Farm. Rabbit chows with and without 0.5% cholesterol and 2% peanut oil supplements were purchased from Harland Teklad.

### Cell culture

Rabbit aortic smooth muscle cells were grown to confluence in 3.5-cm dishes at a seeding density of 4 × 10^4^/ml from cell fractions isolated by collagenase digestion or from explants of normal male New Zealand White rabbit thoracic aorta fragments [7]. Cells were further cultured in Dulbecco's modified Eagle medium (DMEM) containing 10 % fetal bovine serum (FBS) in 5% CO_2 _chamber until about 1.4 × 10^5 ^cells/dish were reached. The absence of alkaline phosphatase activity and the presence of NTP pyrophosphohydrolase activity of cell homogenates were consistent with previous observations with either tissue homogenates or collagenase digests [7,18,19,21,22]. For calcification assay, the Ca × P ion product in DMEM was modified to various levels by adding small aliquots of 100 mM stock solutions of CaCl_2 _and phosphate buffer at pH 7.5 to the media. The endogenous concentrations of both Ca and P in the media and serum were taken into consideration during the adjustment on the basis of the following information. DMEM from the supplier contains 1.8 mM Ca and 0.9 mM P corresponding to a Ca × P ion product of 1.62 mM^2^. A 10 % FBS contributed about 0.18 mM of Ca ions and 0.09 mM of phosphate ions to the culture media. The confluent cells were washed twice with Hank's buffer and then exposed to a 2.5-ml DMEM adjusted to a Ca × P ion product range of 4.5–9.4 mM^2^. Stock solutions of CaCl_2 _and sodium phosphate buffer at pH 7.5 were prepared fresh and filtered through 0.02 μ-filters for sterility and the exclusion of calcium carbonate particles that could be formed upon prolong storage. To prevent pH fluctuations during storage, DMEM were sealed tight with parafilm during the storage and kept in the refrigerator to minimize the loss of bicarbonate resulting from hydrolysis. The media were discarded after 1 week of storage. Sterile tubes with 2.5 ml media in each tube were kept in 5 % CO_2 _for 24 hrs prior to the calcification assay. The change in oxygen tension and osmalality as a result of serum depletion were neither measured nor adjusted.

### Alizarin red staining for mineral deposits

After cells were exposed to modified DMEM with or without FBS for 2 hrs, the media were removed. The cells were washed twice with PBS (phosphate buffered saline) and then twice with distilled and deionized water. Unless stated otherwise, the initial Ca × P ion products of 4.6–9.37 mM^2 ^were present in the media. A 2.5 ml of 1% alizarin red (AR) solution was then added to each dish. After 1 min, the dishes were washed twice with water, once with PBS, and photographed. Under these experimental conditions, the control cultured dishes, which contained cells exposed to culture media with 10% FBS were faintly yellowish and did not exhibit red stains. In contrast, the cells exposed to serum-free media showed bright red stains after 2 hrs of incubation.

### Quantification of mineral deposited by cultured cells

After confluent cells were exposed to modified DMEM with or without serum and different Ca × P ion products for various time intervals, the cells were washed twice each with 2 ml of TBS (Tris-buffered saline, pH 7.6), followed by scraping the cell layers with 0.5 ml of 0.1 N HCl, and then mixed thoroughly using Pasteur glass pipets until the cells were finely suspended. A 10-μl aliquot of the extracts was then taken for protein determinations using a BioRad assay kit. The remaining extracts were centrifuged at 10,000 rpm in a microfuge for 10 min to collect supernatants. Aliquots of 1/5 of diluted supernatants were then taken for the determination of Ca and P content in the supernatants using a commercial Arsenazo III kit (Fisher Scientific) [31] and a standard molybdate-phosphate complex assay procedure (Sigma Diagnostics, Inc.), respectively.

### Fourier transform infrared microspectroscopic analysis

The Ca × P ion product of DMEM was initially adjusted to 9.37 mM^2^. After 2 hrs of exposure to the modified culture media with or without the serum, cells were washed twice with TBS and twice with water and then scraped off from the dishes and finely suspended with Pasteur pipets. Five-μl aliquots (about 1 μg proteins) of suspensions were then spotted onto BaF_2 _windows for infrared analysis. The infrared spectra were obtained by scanning the deposits over a 0.6–0.8 cm path. The wavenumber at 1650 represents the protein amide peak whereas the wavenumbers at 1050–1080 indicate the mineral P phase. A Shimadzu Fourier-transform infrared microspectroscopy model AIM8400/8800 was used for analysis.

### Membrane translocation assay

The assay was performed using a commercial apoptosis kit (Accurate Chemical Scientific Corp.), which measured the uptake of a specific dye by cells as a result of apoptosis-induced membrane phosphatidylserine and phosphatidylcholine translocation [24]. The procedure was used to monitor membrane translocation as a result of serum depletion in cell culture. The assay was performed in 96-well microtiter plates. Cells were grown to confluence and followed by a thorough rinse with PBS to remove serum, and then exposed to media with or without 10% serum for 1 1/2 hrs. At the end point, each well was washed with PBS and then exposed to an apoptosis dye reagent in DMEM with or without 10% FBS for 30 min. The cells were then photographed after the removal of dyes and followed by PBS rinsing. For quantitative assay, the dye was solubilized with the provided detergent and then read at 550 nm in a BioTek EL 800 reader.

### Mass spectroscopy procedure

For spectrometry analysis, calcifying vesicle fractions at protein concentration of 0.5 mg/ml were centrifuged at 250,000 × g for 20 min and then re-suspended in aliquots of water equal to the original volume of fractions. An equal volume of 1 % deoxycholate was added and mixed vigorously. After centrifugation, the precipitates were re-suspended in an aliquot of water. Proteins were precipitated by adding excess volume of alcohol. The precipitated proteins were then solubilized in small aliquots of water and then subjected to 4 M guanidine chloride denaturation and proteolysis by trypsin. Following trypsin digestion, each sample was pressure loaded on a C18 reverse phase nano-column (75 μm ID fused silica packed in-house with 9 cm of 100 Å, 5 μ, Magic C18 particles, Michrom Bioresources). Following a wash with 0.1% formic acid for 15 minutes at 0.5 μl/min, the column was mounted on the electrospray stage of an ESI IT FT ICR mass spectrometer (LTQ FT, ThermoFinnigan) and the peptides were separated on-line with a Surveyor LC with a 0 – 90 % acetonitrile gradient in 120 minutes at an approximate flow rate of 0.3 μl/min. An electrospray voltage of 1.9 kV was used, with the ion transfer temperature set to 350°C. The mass spectrometer was controlled by the Xcalibur software to perform continuously mass scan analysis on the FT followed by MS/MS scans on the ion trap of the six most intense ions, with a dynamic exclusion of two repeat scans of the same ion, 30 s repeat duration and 90 sec exclusion duration. Normalized collision energy for MS/MS was set to 35%. For data analysis all MS/MS scans were searched using the Sequest algorithm included in Bioworks 3.1 (ThermoFinnigan) using a rabbit database derived from the NCBI Nr (2006-07-08) repository.

### Statistical analysis

Student's t test was used to compare the significance of the mean differences between control and experimental groups. One-way ANOVA was used to compare the significance of the differences among 3 or more groups. Three replicate experiments were used for each set of tests. For each experiment, duplicate determinations for Ca and P determinations were averaged prior to statistical analysis. ANOVA analysis was initially done globally for overall estimates and followed by Tukey analysis. A Prism software program was used for statistical analysis.

## Competing interests

The author(s) declare that they have no competing interests.

## Authors' contributions

HHTH was responsible for experimental designs, data interpretations, and execution of experiments including cell culture and calcification. AA and MTV are responsible for proteomic projects.
